# The role of motivation in the diffusion of innovations in Canada’s long-term care sector: a qualitative study

**DOI:** 10.1186/s43058-020-00069-7

**Published:** 2020-09-24

**Authors:** Lauren MacEachern, Lisa Cranley, Janet Curran, Janice Keefe

**Affiliations:** 1 Institute for Health Policy, Management & Evaluation, University of Toronto, 155 College Street, Suite 425, Toronto, Ontario M5T 1P8 Canada; 2 Lawrence S. Bloomberg Faculty of Nursing, University of Toronto, 155 College Street, Suite 130, Toronto, Ontario M5T 1P8 Canada; 3 School of Nursing, Dalhousie University, 5869 University Avenue, Room 121, Halifax, Nova Scotia B3H 4R2 Canada; 4 Nova Scotia Centre on Aging, Department of Family Studies and Gerontology, Mount Saint Vincent University, 166 Bedford Highway, Room 202, Halifax, Nova Scotia B3M 2 J6 Canada

**Keywords:** Long-term care sector, Motivation, Opinion leadership, Diffusion of innovations, Professional advice seeking networks

## Abstract

**Background:**

Long-term care facilities offer shelter and care for Canadian seniors; however, there are great variances in the quality of care that is provided to older adults across facilities. One factor that could contribute to this variation in quality is the diffusion and implementation of advice and innovations within this sector. This study sought to understand the motivations of identified opinion leaders within the Canadian long-term care sector to disseminate advice within their social networks. Research questions addressed specific drivers of motivation and the potential outcomes of having motivated opinion leaders present within interpersonal advice-seeking networks with respect to diffusion and implementation of innovations in the Canadian long-term care sector.

**Methods:**

This secondary analysis study analyzed semi-structured qualitative interviews with opinion leaders (*n* = 13) and advice seekers of opinion leaders (*n* = 13) from a national, social network study, *Advice Seeking Networks in Long Term Care* (Cranley et al. 2019; Dearing et al. 2017). Constant comparison analysis was used and supported by a theoretical framework developed from diffusion of innovation theory and the COM-B framework.

**Results:**

The motivations of opinion leaders in the Canadian long-term care sector were represented across seven themes: obligations of the position, value of education, systemness, relationships, supportiveness, passion, and caring nature.

**Conclusions:**

This research provides further evidence that opinion leaders in the long-term care sector are motivated individuals and that they are using this motivation as a driver to create change and improve care practices. As residents of the long-term care sector continue to increase in number and complexity, the presence of motivated opinion leaders represents a promising outlook for the future through achieving specific outcomes such as the diffusion and implementation of innovations, an increased sense of community within the network, and increased readiness for the future.

Contributions to the literature This paper provides:

- An enhanced understanding of the drivers of motivation for opinion leaders in Canada’s long-term care sector
- An enhanced understanding of opinion leader characteristics
- Specific examples of successful diffusion/implementation projects facilitated by opinion leader involvement in the Canadian long-term care sector

## Background

Long-term care facilities (LTCFs) offer an integral option for care to Canadian seniors that is recognized within the care continuum [1, 2]. While LTCFs across Canada are guided by various structures of ownership and different models, levels, and philosophies of care [1], the provision of quality care should be recognized within each facility as the primary driving factor. However, there are great variances in the quality of care that is provided to older adults. There are many factors that could contribute to this variation of quality; one pertains to the ways in which advice and innovation is diffused within the long-term care sector and if or how that advice and innovation is subsequently implemented [3].

As population aging continues to increase in Canada, the demand for long-term care services and placement in LTCFs increases in tandem [4, 5]. In addition to higher consumer demand, LTCFs are also experiencing a greater challenge with the complexity of residents’ needs [1, 2]. Implementation of knowledge derived from research within LTCFs has never been more critical; however, a substantial lag has been recognized between the production of research and its implementation within this practice setting [1, 6–9]. The time for adoption of best practice programs and innovations to increase quality of care within the long-term care sector is now [1, 6].

### Study rationale and purpose

When implementing a change in program or culture in any organization, buy-in from those in positions of leadership and management is of the upmost importance [10–12]. In LTCFs, this position is often held by directors of care or directors of nursing. Decision-makers are highly influenced by individuals in their professional networks and these relationships of advice seeking and advice giving can be observed through social network analysis studies [10]. Through social network analysis, we are able to identify key players in advice-seeking networks including advice seekers, boundary spanners, and opinion leaders [8]. Opinion leaders are defined as individuals in a particular field with the ability to influence the opinions and decisions of others and are influential in establishing buy-in [13–15]. With this ability, knowledge translation techniques have been developed to harness the qualities and characteristics of opinion leaders as a targeted implementation strategy for knowledge diffusion [15, 16]. With the ability to expedite the diffusion process, opinion leaders are critical to the healthcare sector and present a promising impetus to the implementation and adoption of practices that are evidence-based and community-oriented [16, 17].

In research fields of behavior change and organizational change, motivation is recognized as an important driver for workplace improvements; however, there is a paucity of research in this area with respect to the long-term care sector [11, 18]. Further investigation of the motivation of opinion leaders and other related factors such as capability and opportunity is important to understand the influence that these factors have on the decision-making processes by directors of care. This understanding could lead to an increased capacity to tailor implementation strategies and help to ensure that the long-term care sector is successfully prepared to accommodate the demands of our aging society.

A two-phase social network analysis study was completed to provide an “outside-inside” view of the advice-seeking behaviors of decision-makers in the Canadian long-term care sector using quantitative and qualitative approaches [19, 20]. This secondary analysis study elaborates on previously reported qualitative results by Cranley and colleagues [19], which identified the motivations for seeking and providing advice as a key theme. The aim of this study was to determine how the presence or absence of opinion leader motivation, with consideration for associated factors of capability and opportunity, impacts the diffusion and/or implementation of advice within the Canadian long-term care sector. To determine this impact, the following questions were addressed: What are the drivers of capability, opportunity, and motivation for opinion leaders within the interpersonal advice-seeking networks of this sector? What are the potential outcomes of having motivated opinion leaders within the long-term care sector with respect to the diffusion and implementation of innovations?

## Methods

### Study population

The study sample was selected exclusively from the original 39 telephone interview respondents of the social network analysis study, *Advice Seeking Networks in Long Term Care* [19, 20]. With an interest in the role of opinion leaders and advice seekers, the subset of interviews conducted with 13 identified opinion leaders and 13 advice seekers of opinion leaders (*n* = 26) were purposefully selected for secondary data analysis. Participants in these interviews were from the provinces of Nova Scotia, New Brunswick, Prince Edward Island, Manitoba, Saskatchewan, Alberta, British Columbia, and the Northwest Territories. Interviewees from both subsamples (opinion leaders and advice seekers) were identified separately and were not part of a matched dyad.

### Data collection and measures

In phase 1 of the primary study (Fall 2014), senior leaders in LTCFs in 8 Canadian provinces and 3 territories were targeted based on their decision-making role in matters concerning resident care. In a quantitative online survey, LTCF senior leaders were asked to list the individuals external to their facility whose advice they seek or behavior they monitor about delivery of quality care, care improvement, and innovation. Through this survey, key players within the Canadian long-term care sector were identified, including advice seekers, opinion leaders, and boundary spanners, based on their social network analysis scores of in-degree centrality^1^ and betweenness centrality^2^. Quantitative results were visually presented using social network maps [20].

In phase 2 of the primary study, qualitative semi-structured interviews were conducted to bring better understanding and context to the survey results. The interviews were conducted over the telephone with a purposively selected subsample of 39 participants from phase 1 based on their network scores of in-degree centrality and to ensure a sample representative of the provincial ratios was reflected (i.e., provinces with a higher population had more interviewees). Interviews were conducted over the phone rather than in-person for feasibility and to facilitate a pan-Canadian perspective. The interview process took place between Fall 2015 and Spring 2016, with the interviews lasting an average of 38 min (range 18–74 min) [19]. Each interview was conducted by a research assistant and senior researcher, audio-recorded, and then transcribed verbatim.

### Secondary analysis

**Fig. 1:**
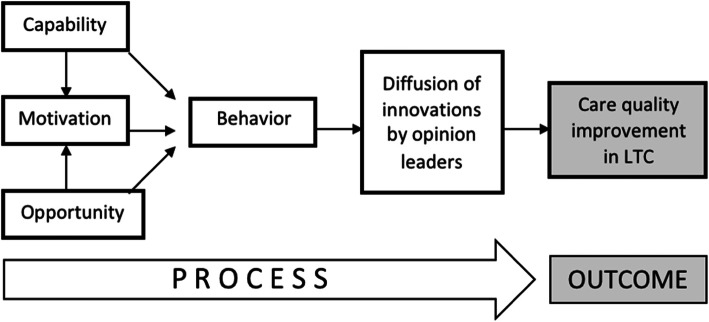
Theoretical framework

This paper presents a secondary analysis of the qualitative interviews with opinion leaders and the advice seekers of opinion leaders from phase 2. The original interviews were conducted to elicit an “insider view” of the networks through an exploration of the nature of advice relationships, characteristics of network participants, and types of advice that were shared and received [19]. This secondary analysis study sought to determine the role of motivation, with consideration for the associated components of capability and opportunity, on the diffusion of advice within the long-term care sector. With a goal of expanding and deepening the existing knowledge generated from the primary study [19], the present study built on the findings from the primary interview analysis through further exploration of the identified motivational characteristics, as they relate to diffusion of innovation theory and the COM-B framework. Diffusion of innovation theory and the COM-B (capability, opportunity, motivation, behavior) framework were used as guiding theoretical frameworks for this work [14, 18], as diffusion of innovation theory describes how innovative ideas and practices are diffused within a social network [14, 22, 23] and the COM-B framework considers the behavior of an individual with respect to three interactive components: capability, opportunity, and motivation (Fig. 1) [18].

Secondary data analysis of the 26 selected transcripts was conducted using techniques borrowed from grounded theory [24] using NVivo 11 software. Constant comparison analysis from grounded theory was used in this study, with each emerging incident and thought being compared to those previously realized [24, 25]. Each interview was coded by the lead author (LM) with regular conversations with a second author (JK) to discuss findings, interpretations, and thematic development. When areas of misalignment were encountered, the other authors (LC and JC) with extensive knowledge of the transcripts and theoretical framework were consulted. While the opinion leader interviews were the primary source of data used in answering the research questions of this study, the advice seeker of opinion leader interview contributed complementary knowledge from a different perspective. The advice seeker of opinion leader interviews were used to provide examples and statements as recipients of the advice.

While ethical approval was obtained from all participating universities in the primary study, ethical approval for the secondary analysis was obtained through the University Research Ethics Board at Mount Saint Vincent University.

## Results

### Interview respondents

Opinion leaders and advice seekers of opinion leaders whose interviews were analyzed in this secondary analysis study were located in Nova Scotia, New Brunswick, Prince Edward Island, Manitoba, Saskatchewan, Alberta, British Columbia, and Northwest Territories [19]. The complete demographic and employment characteristics of opinion leaders are summarized in Table 1. The opinion leaders occupied various positions, including director of care and clinical nurse specialist in a LTCF, and liaison officer, senior nurse consultant, and director of continuing care at a regional or provincial level.

**Table 1:** Demographic and employment characteristics of opinion leaders and advice seekers [*N* (%), unless otherwise stated]

	Opinion leaders	Advice seekers
Respondents	13	22
Gender		
Women	13 (100)	21 (95)
Men	0	1 (5)
Age		
20–39	1 (8)	1 (4.5)
40–59	11 (85)	20 (91)
60+	1 (8)	1 (4.5)
Professional role		
Senior leadership position in an LTCF	1 (8)	22 (100)
Corporate-level position in LTC organization	2 (15)	0
Position in regional health authority/government	10 (77)	0
Education		
Diploma/certificate	1 (8)	9 (41)
Bachelors	6 (46)	8 (36)
Graduate	6 (46)	5 (23)
Professional background		
Nursing	12 (92)	18 (82)
Business	1 (8)	2 (9)
Other	0	2 (9)
Years worked [*M* (*SD*)]		
In long-term care	16 (14)	15.52 (9.98)
In current position	6 (5)	6.82 (5.40)

Demographic information for all advice seekers was pooled (i.e., advice seekers of opinion leaders (*n* = 13) and advice seekers of boundary spanners (*n* = 9))

### Themes

Motivation was the primary COM-B component of interest in this analysis. The motivations of opinion leaders to give advice and diffuse innovations within the long-term care sector were driven by factors identified across seven themes, with consideration for additional contributing factors such as capability and opportunity. The seven motivational themes of opinion leaders were: obligations of the position, value of education, systemness, relationships, supportiveness, passion, and caring nature. The following sections described these themes in more detail, with descriptions of how factors of motivation, opportunity, and capability emerge within each.

#### Obligations of position

One dominating motivator for opinion leaders in providing advice or sharing innovations within the long-term care sector was the obligations of a professional position. While most opinion leaders spoke of this motivator in combination with other underlying components, the obligations of their contract or job description/responsibilities was discussed by 12 of the 13 opinion leaders as a driving component to providing advice within the sector. Many advice seekers of opinion leaders also referenced the position of the opinion leader as the main characteristic that distinguished them as a key source of advice.

“So yes, [sharing advice] is a part of my position as a senior nursing consultant for long-term care. That’s what I do provide programs, planning, support, you know, if they’re asking something that’s relevant to the setting or the resident or… best practice, clinical practice, then definitely - well, that’s part of what my role is”—opinion leader from West/North

As part of their position, five opinion leaders spoke about the opportunity of having access to numerous LTCFs and their obligation to provide advice and knowledge to these facilities on a routine basis. Opinion leaders described their networks as an extension of their position, following a natural systematic flow that was less based on personal characteristics but rather professional aspects.

“We see it as our responsibility to disseminate best practice evidence and information that comes to us.”—opinion leader from West/North

#### Value of education

Opinion leaders discussed their continued interest in capacity development and appreciation for continued educational opportunities offered to them due to the nature of their position, including attendance at workshops and conferences. The benefits accrued from participation in such opportunities are not lost on the opinion leader, but rather harnessed as a motivational driver to share knowledge with other decision makers and healthcare staff.

While opinion leaders are motivated to share their own knowledge, they also promote opportunities for others to further develop knowledge and skills in elder care. Opinion leaders recognize the long-term care sector as an evolving and dynamic work environment that is often challenged with limited resources, thus increasing the importance of continuous education to ensure best practices are up to date and available resources are used most effectively and efficiently.

“We know that things are changing rapidly, so we have to constantly be keeping ourselves current and look at ah… and we also know that you know there’s been and continues to be pressure on the resources that are available to deliver programs.”—opinion leader from West/North

Opinion leaders in the long-term care sector have built their capabilities for advice and knowledge sharing through numerous endeavors, including having worked for many years and in many different positions within the health care sector. These capabilities were echoed by the advice seekers of opinion leaders in their respective interviews, with experience, knowledge, and credibility identified as characteristic traits enabling an opinion leader to stand out in their position.

#### Systemness

Opinion leaders are also motivated by a desire to improve care quality in the long-term care sector. This could be described as a sense of “systemness,” which is defined as a feeling or sense of accountability and responsibility for long-term care sector improvements [19]. For example, opinion leaders are motivated to provide advice or knowledge on innovations with a goal to ultimately improve the quality of resident care in LTCFs. When describing their motivation to share advice, one opinion leader states:

“But also, fundamentally, that’s my value, is that I want to be able to provide the best care that we possibly can. And I think that that’s kind of probably why I’ve grown into this role that I have, is because I’m able to provide that leadership.”—opinion leader from West/North

Additionally, opinion leaders are motivated to share advice and innovations within the sector to improve the working conditions and safety of healthcare staff. Some opinion leaders drew motivation from their own previous experiences working as healthcare staff in LTCFs and spoke of this as a motivational driver to continuously increase the safety standards for the staff of their LTCFs.

“Because we want what’s best for the resident. And I want what’s best for our staff. And I want there to be a healthy, home-like environment for those we care for. And I want there to be a safe working environment, and a healthy environment for my staff.”—opinion leader from West/North

#### Relationships

Opinion leaders value their relationships with others in the sector, both long-standing and evolving. They are motivated by these relationships because they wish to maintain them, they respect the other individual in the relationship, and they feel a sense of comfort and teamwork from the relationships they have developed over time.

“I do have a good personal relationship with a lot of the directors of nursing, and I’ve actually developed personal relationships with some of the social workers as well as some of the nurses that have been in touch with me. And so that motivates me too, because I really respect the work that they’re doing, and I respect them as professionals and as individuals.”—opinion leader from West/North

There were a few examples in which the advice seekers spoke to the relationships they had developed over time with opinion leaders. While these relationships were valuable in themselves, advice seekers also acknowledged benefiting from the large and diffuse networks of the opinion leader. Advice seekers also described opinion leaders as being present and available, which could contribute positively to the development and sustainment of relationships and the diffusion of innovations:

“He always put himself out there to the forefront, to say, ‘anything at all - give me a call’”—advice seeker of opinion leader

One way in which opinion leader created relationships was through their participation in committees and groups within the long-term care community. The importance of participating in committees and group meetings as an opportunity for networking and knowledge sharing and seeking was evident in the interviews. Eleven of the 13 opinion leaders spoke of group meetings and committees of which they were a part that are organized at a local, provincial, regional, and national level. These meetings are formed to discuss various topics of interest related to long-term care policy, care improvement initiatives, and general knowledge and advice sharing.

“I’ve been quite involved in the [provincial gerontological nursing association]. So that’s another really valuable way of networking and developing some connections. And knowing what’s going on in gerontology and seniors’ health caring practice.”—opinion leader from West/North

#### Supportiveness

Opinion leaders are driven by the prosocial characteristic of wanting to make the lives of others easier. This trait could be described as a sense of supportiveness, where the opinion leaders care for those working in the long-term care system and share advice and innovations to make the lives of their colleagues easier and ultimately improve care or quality of work life.

“I never thought about what is in it for me. I guess I look at it that, if I can make somebody’s life easier, then great. If I can improve quality of care in my own home or another home, then that’s great”—opinion leader from Atlantic

Furthermore, two opinion leaders spoke specifically about the importance of providing coaching and mentoring to those who seek their advice, allowing their mentees to work through challenges and understand the rationale behind the solutions, rather than just providing them with a yes or no answer. It is clear they are invested in the long-term care sector and care greatly for its continued success with future generations of leaders.

“There’s an element of coaching and mentoring in there as well. So, instead of just making a decision, not really explaining it, having a little bit of that banter back and forward so that they can truly understand and challenge me as well. […]If it’s something that has a little bit of negotiation, I really try to foster that coaching and mentoring as well.”—opinion leader from Atlantic

#### Passion

Opinion leaders are highly motivated by their passion for long-term care. In this study, passion is described as an extension of an innate characteristic that has developed into an interest or personal investment. Opinion leaders are passionate about the care they provide and they have a personal investment in their work and the goals they set to achieve through their work. In some of the interviews, the opinion leaders’ passion was driven by a personal experience, feeling, or emotion. The motivation to improve care quality and engage as a leader in the sector in these cases was derived from a personal connection.

“But I also think that it’s because this is my community. These could be my family members. This could be my friends. It’s my friend’s families. It could be me one day, it could be them one day. So, this is my community, this is my home. So whatever happens here, it’s because this is our home. And we deserve this as we age. So I need to support that we’re doing it right now.”—opinion leader from West/North

Advice seekers also identified the passion of opinion leaders as a driving motivator for sharing advice and relationship building within the sector.

“I think her passion for long-term care. She has a genuine passion for the residents that we care for. And that really stands out and… has always stood out for me. That if somebody has the passion, then they’re the person that I want to talk to”—advice seeker of opinion leader

#### Caring nature

Opinion leaders described their connection to the long-term care sector and those who work and live within it as an inherent characteristic or being “part of their nature.” Some opinion leaders were naturally and genuinely driven by their caring nature to diffuse innovations within the sector, rather than being nurtured to take on such duties. Personal characteristics of opinion leaders, such as personality, were recognized by advice seekers as a characteristic that drives the opinion leader’s ability to be effective in this informal sector role. It was also observed that a background in nursing was associated with the theme of caring nature and used as the rationale to explain such an innate characteristic.

“I’m a nurse in my background. So it’s just that caring, helping nature, right? Like my interest in helping. And I really like problem-solving. And that kind of thing. So I would say it’s part of my natural probably instinct as well.”—opinion leader from West/North

#### Outcomes of motivation

With a greater understanding of the motivation that drives an opinion leader’s behavior to share advice within the long-term care sector, we may start to consider the outcomes or impact of this behavior. This section addresses the study’s second research question by providing specific examples of innovations that were diffused or implemented by an opinion leader within the network. Twelve of 13 opinion leaders provided at least one example of an innovation they shared or helped to implement with the intention of improving care practices. Examples of these innovations included LTCF policies regarding restraint use and responsive behaviors, communication boards for use at the resident’s bedside, and information around standardized clinical definitions for infections.

Opinion leaders often spoke about their innovative endeavors with pride, ownership, and confidence. They recognized each implementation effort as an opportunity to gain further education and experience in care improvement for both themselves and those involved. By undergoing this process of knowledge sharing and implementation on different occasions, opinion leaders indicated that they have increased knowledge into the conditions needed for successful diffusion, implementation, and adoption. Opinion leaders invested time and effort in the diffusion and implementation process because they were motivated by the outcomes they knew could be achieved through evidence-based approaches to care improvement.

“The iPod project, for one. […] [I] wanted to share it, just because of the importance of music, and you know, being at a conference, that information that I had received from different sources, one being the [institute], one being [LTCF], who started the iPod project in [province]. And took that back to my homes that weren’t able to be there, and send that out. Send them videos, clips of music and memory. I just felt it was so important and had seen the… anecdotal results of it were very positive. So I shared it with the homes. I shared it with the directors of care. I share it with the administrators, activity people. On inspection – that’s kinda one thing that I took to all my inspections that year. […] And it had quite a positive impact.”—opinion leader from Atlantic

In these examples, the primary outcome of motivated opinion leaders was demonstrated: the diffusion and implementation of innovations within the long-term care sector. Additionally, the advice-sharing behaviors of opinion leaders have helped to establish a sense of community within the sector through personal and professional relationship building. With increased knowledge sharing and innovation diffusion, opinion leaders also spoke of preparing the next generation of decision-makers to enhance the success of the sector. They recognized the challenges that the long-term care sector will have to overcome in the present and future, and they were motivated to do their part in seeing its success.

## Discussion

As the Canadian population of older adults continues to increase, the need to continuously improve the quality of care provided in LTCFs increases in parallel. Such improvements may be initiated and supported through the use of advice-seeking networks to share knowledge and innovations throughout the long-term care sector. Opinion leaders play an integral role in this process, as they are recognized by their peers as the key sources of advice within the sector [15]. The purpose of this research was to examine what motivates opinion leaders to diffuse advice within the advice-seeking networks of Canadian LTCFs and to determine the outcomes of that motivation from their perspective.

### Drivers of motivation: from professional to prosocial

Previously conducted research has indicated that motivation can be influenced by and drawn from many sources based on situational context and that these sources can change over time [26]. The findings of this study support this understanding, with seven motivational drivers emerging from the interviews with long-term care opinion leaders: obligations of the position, value of education, systemness, relationships, supportiveness, passion, and caring nature. Each opinion leader within this dataset was motivated by a source within at least one of the identified themes, indicating that the absence of motivation was not detected.

**Fig. 2:**
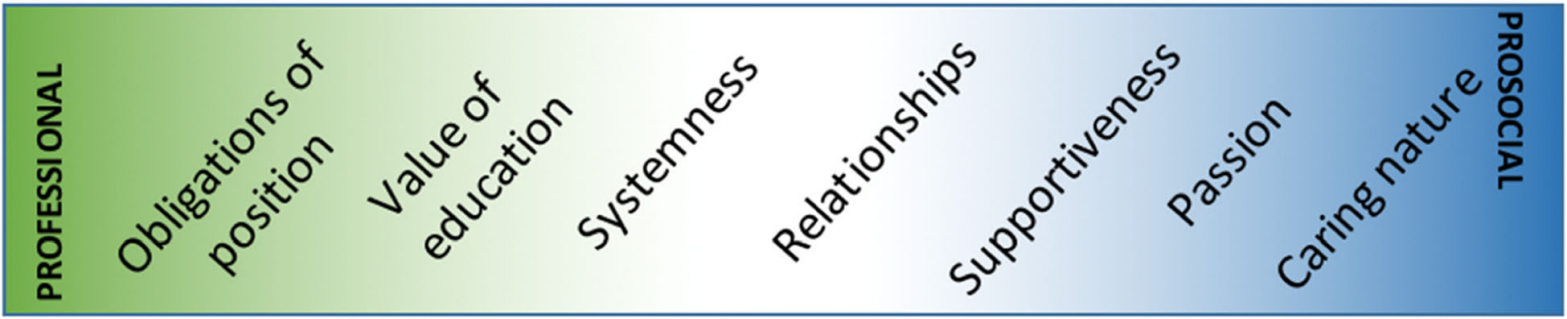
Motivational scale

Figure 2 presents the motivational drivers identified in this study as they may be understood on a sliding scale from professional to prosocial. The components of this motivational scale are fluid and may “slide” or interchange with one another, allowing for the heterogeneity of opinion leader motivation to be expressed on an individual level. In a comparable study of interest, de Guzman and colleagues [27] discuss geriatric service motivation typologies to explain the motivation and attitudes of Filipino nurses caring for the older adult population. These mutually exclusive typologies included “single loop motivation” and “double loop motivation,” which describes nurses who are driven by their perceived call of duty and nurses who are influenced by their history and an ultimate sense of joy and fulfillment, respectively [27]. In this comparison, professional motivators could be associated with single-loop motivation and prosocial motivation could be associated with double-loop motivation. de Guzman [27] found that nurses were most likely to be driven by professional factors pertaining to self-betterment and role obligations at the commencement of a career in geriatric nursing; however, as time passed, they were more likely to be motivated “from the heart.”

Previous research has suggested that prosocial and intrinsic motivators were the key drivers for individuals in a caring profession [26, 28]. Motivation discussed within published literature identified only behaviors in relation to the nature of their work, but did not address the diffusion of innovations specifically. Based on this, it was expected that prosocially and intrinsically driven factors would arise more prominently within the results of this study. However, the most prominent motivator for opinion leaders was the obligations of their professional position, which could be interpreted as an extrinsic motivator [28]. This factor was also highly impacted by the associated factor of opportunity, as evaluated using the COM-B framework [18]. Opinion leaders discussed a significant opportunity to diffuse innovations within a specific group of individuals that arises from the connections within their position, including other facilities under the same ownership or jurisdiction. Due to the nature of these connections, innovations are diffused effectively and strategically, often with the desire to standardize care practices and prevent a phenomenon referred to by many as “re-creating the wheel.”

At the balancing point of the motivational scale lies the motivational driver of systemness, which impacts care quality improvement and quality of work life improvement for healthcare staff within LTCFs [19]. This motivational factor was frequently discussed by opinion leaders; however, the ways in which it was discussed emerged differently. Systemness as a motivational driver was often discussed in association with the obligations of their position, as continued improvement to such aspects of quality was important to the well-being and reputation of the facility as a whole. In contrast, components of prosocial motivation emerged as an additional consideration. With the interests of others at first mind, opinion leaders wish to provide the best possible care for the residents and the best quality work life for care staff.

Many opinion leaders contributed clear statements of “the desire to expend effort to benefit other people” [26]. At times, this was accompanied by a feeling of personal satisfaction attained by helping others, or intrinsic motivation [19]; however, the majority of statements demonstrated the influence of prosocial motivation as a driver to diffuse advice and knowledge within the long-term care sector. While the theme of systemness persists as an underlying driver of these statements, the themes that fall to the right of the motivational scale truly exemplify the notion of prosocial motivation. Prosocially motivated individuals are said to be driven by an interest in the outputs for the individuals targeted with their work, as well as a sense of duty to protect and promote the well-being of others [26]. Additionally, prosocial motivation drives a sense of focus to the future [26], in this case the future of long-term care. It was clear from the opinion leader interviews that motivation from a place of passion and caring nature has great impact on the diffusion of innovations within the sector. Advice seekers recognized the passion that opinion leaders hold for the long-term care sector, which impacted their likelihood to seek advice from this individual above others. Linked with these statements of caring nature and passion, some of the opinion leaders with a professional nursing background used this characteristic as an explanation for the passionate and caring nature that guides their work in the long-term care sector. It is understood within the literature that nurses are not only driven from a place of prosocial motivation when choosing to embark on a career in this field, but also influenced by this driver as a source of motivation for continued success and career commitment [29]. The findings of this study are found to align with this understanding, as many opinion leaders within the Canadian long-term care sector, with and without a background in nursing, were influenced by characteristics of prosocial motivation.

### Outcomes of motivated opinion leaders in the long-term care sector

There are several outcomes that emerged from the presence of motivated opinion leaders in the long-term care sector. The first is evidence of diffusion and implementation of innovation. As indicated by Rogers [14], the involvement of opinion leadership within the diffusion and implementation process is likely to result in a more successful outcome. There are many reasons for which this relationship between opinion leadership and successful diffusion and implementation may exist. Opinion leaders bring years of experience and often a varied perspective to implementation after having worked in different sector positions [13, 14, 30]. With constant consideration for care quality improvement, the opinion leader characteristics of being future-focused and outcome-oriented is important to the diffusion and implementation of innovations. Opinion leaders represent a strong presence within their communities, particularly at the local level and are able to tap into network resources to affect change in a way that is credible and convincing [14, 15].

Evidence of innovation diffusion as an outcome of opinion leader motivation was particularly important in this study. Due to the nature of secondary data analysis, there is insufficient evidence to argue that advice seekers contribute to the diffusion and implementation of advice and innovations. There is sufficient evidence, however, based on the results of the network analysis component of this project, to suggest that the identified opinion leaders take part in such behavior more frequently and to a larger audience [20]. Future research may further explore the differences of advice seeker and opinion leader roles in the process of innovation diffusion.

## Limitations

When using data for secondary analysis, there are key considerations related to data collection and analysis that may present as potential limitations. To overcome potential limitations in the interpretation of data, raw audio recordings and transcripts of the interviews were used during analysis. Additionally, consultation with the original interviewers was sought throughout the data analysis phase. Differences in the research questions and aims of the original study and the secondary study could be considered a limitation when conducting secondary analysis; however, the authors in this study observed sufficient alignment between the original objectives and the novel focus on motivation. Additionally, a finite dataset may present potential barriers in reaching theoretical saturation; however, analysis was continued until the study’s research questions were addressed and data saturation was reached [25].

Only one participant in this data set for secondary analysis identified as male. While the population of those employed within the Canadian long-term care is highly female-dominant, this could lead to bias within the results of our study. Further research may seek to explore the advice-seeking behavior of male employees within the long-term care sector in greater detail.

## Conclusion

This research provides evidence of motivated opinion leaders within in the Canadian long-term care sector and an understanding that this motivation is being used as a driver to create change and improve care practices. While the findings of this study are based on one group of individuals at one snapshot in time, it is possible that such observations may be transferable to those in similar roles over time. This has important implications for the diffusion and targeted dissemination of policy and practice changes throughout the Canadian long-term care sector and contributes to our conceptual understandings of opinion leader characteristics within the field of knowledge translation and dissemination science. As residents of the long-term care sector continue to increase in number and complexity, the presence of motivated opinion leaders represents a promising outlook for the future through achieving specific outcomes such as the diffusion and implementation of innovations, an increased sense of community within the network, and increased readiness for the future. Further research may seek to explore approaches to foster opinion leader motivation for advice sharing, as this may represent a critical effort toward overall improvement for care quality in this sector.

## Data Availability

The data supporting the conclusions of this article are housed in the secure and confidential Health Research Data Repository (HRDR) in the Faculty of Nursing at the University of Alberta (https://www.ualberta.ca/nursing/research/supports-and-services/hrdr/research-supports-and-services/hrdr), in accordance with the health privacy legislation of relevant health jurisdictions. Data specific to this manuscript can be requested through the TREC Data Management Committee (joseph.akinlawon@ualberta.ca) on the condition that researchers meet and comply with the Translating Research in Elder Care (TREC) and HRDR data confidentiality policies.

## Abbreviation

LTCFs: Long-term care facilities

## Footnotes

1 In-degree centrality: number of incoming ties received by a node (number of relationships) [21]

2 Betweenness centrality: the degree to which a node lies on the shortest path to others in the network [20]
